# Structural characteristics of periodontal ligaments in the pig model with experimental periodontitis

**DOI:** 10.36879/IJTS.21.000106

**Published:** 2021-12-10

**Authors:** Mojdeh Eftekhar, Lauren Lee, Atriya Salamati, Zi-Jun Liu

**Affiliations:** Department of Orthodontics, University of Washington, Seattle, USA

**Keywords:** periodontitis, periodontal ligament, sharpey’s fibers, alveolar bone, pigs

## Abstract

To investigate how the structure of Sharpey’s fibers in the periodontal ligaments (PDL) were affected by experimental periodontitis in a young pig model, 7 were periodically inoculated with four types of bacteria and a ligature around the last maxillary molar for 8 weeks to induce periodontitis (PG), and 10 served as controls (CG). The harvested molar blocks were sectioned coronally and stained with either hematoxylin & eosin (H&E) or Sirius Red (SR). The H&E-stained images were first reviewed. Then, images of each adjacent SR-stained sections were captured at the region close to the apex of mesial roots under polarizing light microscopy. Sharpey’s fiber numbers in each bundle, total bundle numbers, connection of the bundle to the cementum and alveoli, as well as bundle angulations to the molar long axis were quantified in the defined area 500μm apical to the root apex. Compared to CG, PG showed the following features: 15.13% less total bundle number; 17.8% less bundle size; significantly less connected fiber bundles; 9.2% more interrupted fiber bundles; and 3.85% more oriented toward the cervical in the bundle angulation. These results suggest that experimental periodontitis alters the PDL structure, leading to more interruptions of Sharpey’s fiber attachments to the cementum.

## Background

Periodontitis is an infectious inflammatory disease caused by the bacteria in the dental plaque, resulting in the progressive destruction of the tissues that support the teeth, i.e., the gingiva, periodontal ligament, cementum, and alveolar bone [1]. Its advanced form is characterized by periodontal ligament loss and destruction of surrounding alveolar bone. It is the main cause of tooth loss and is considered one of the two biggest threats to oral health [2].

Increased tooth mobility can occur because of advanced forms of plaque-associated periodontal disease [3]. According to the results of another study, both alveolar bone loss and alteration of periodontal ligament have roles in hypermobility occurrence [4]. Cementum is a calcified tissue that helps in providing an attachment site for PDL fibers to bond with the root surface. It has been shown by certain observations that cemental apposition decreased by aging in periodontally diseased teeth [5].

The sharpy’s fibers are partially composed of mineralized perforating fibers which represent the terminal ends of the PDL principal fibers and help anchor the tooth to the bone [6]. These fibers are in teeth’s alveolar sockets and embedded in both alveolar bone and cementum. The Sharpey’s fibers on the cementum side are thinner and more abundant comparing to the alveolar bone side fibers [7]. Due to the birefringent nature of Sharpey’s fibers, microscope equipped with polarized light can be used to visualize and characterize their structures and orientations. Given the fact that these birefringent fibers are vulnerable to aging and pathological factors [8], the present study was designed to evaluate the size, attachment patterns, and orientations of Sharpey’s fibers in a young pig model with induced experimental periodontitis. It was hypothesized that the structures of Sharpey’s fibers are altered or damaged in a periodontally infected tooth.

## Materials and methods

### Animals

Seventeen 3-months-old juvenile farm pigs (Progressive Swine Farms, Woodinville, WA) were used for the study. The animal protocol was approved by the Institutional Animal Care and Use Committee (IACUC), University of Washington (Protocol# 3393-03).

### Induction and verification of experimental periodontitis

**Figure 1:**
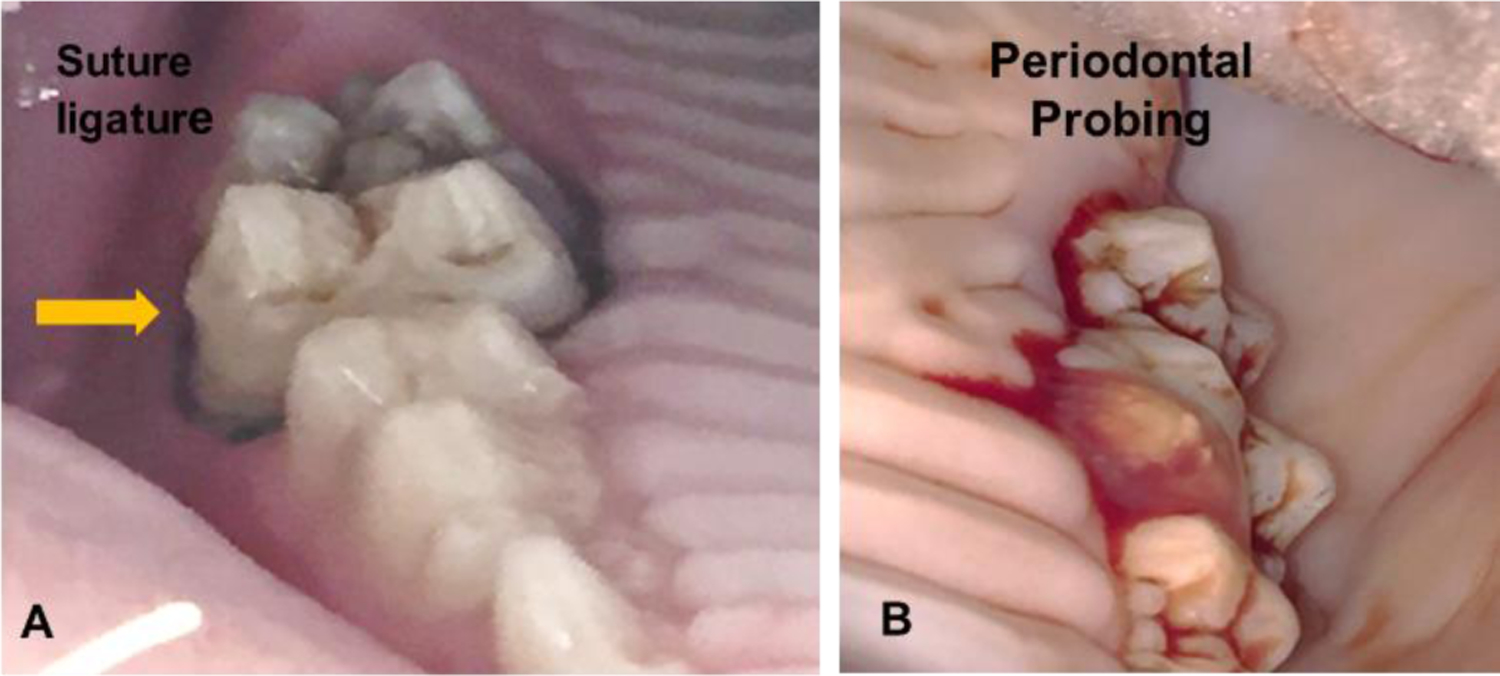
A: Ligature for experimental periodontitis induction (yellow arrow). B: Bleeding when performing the periodontal probing.

Of seventeen pigs, ten were periodontally healthy and served as the control group (CG), and seven were subjected to the induction of experimental periodontal disease (periodontitis group, PG) bilaterally (n=4) or unilaterally (n=3). The induction side was determined by a coin toss. The detailed procedures to induce and verify the experimental periodontics around the last upper deciduous molars were described previously [9]. In brief, under anesthesia, a ligature (3–0 silk braided suture) and overhang composite markers were placed around the molar. The periodontal bacteria cocktail containing the bacteria of *P. gingivalis*, *S. gordonii*, *A. actinomycetemcomitans*, and *F. nucleatum* [10–12] were inoculated around the ligature. The ligature was checked, and the bacteria cocktail was refreshed twice a week. Dental apical radiography (PA), and six-site perio-charting/probing were performed weekly for a period of 8 weeks (Figure 1). Dental plaque and bleeding upon probing were scored. Regular pig chow was offered throughout the experimental period, and the body weights were tracked weekly.

**Figure 2:**
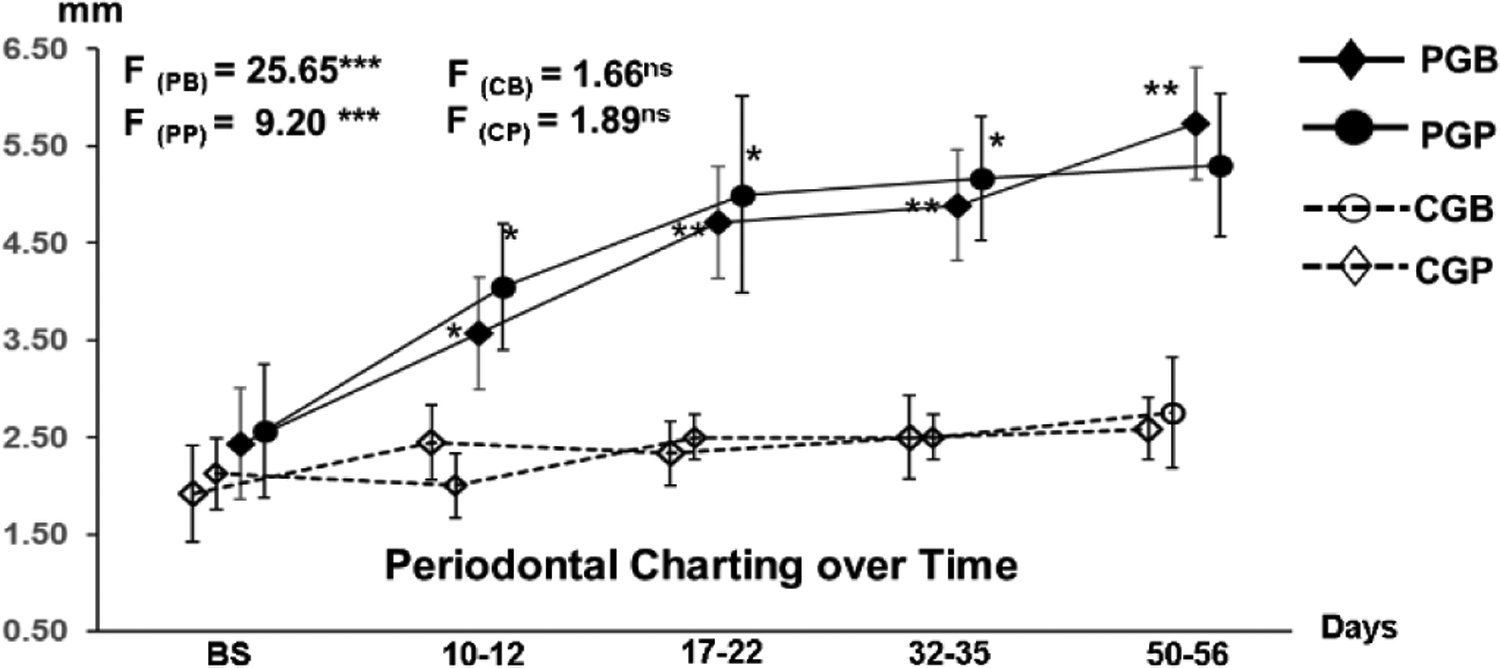
Comparisons of Periodontal pocket depth over the period of 8 weeks. **PGB:** Periodontitis group buccal root. **PGP**: Periodontitis group palatal root. **CGB:** Control group buccal root. **CGP:** Control group palatal root. *: p < 0.05; ** p < 0.01; ***: p < 0.001.

**Figure 3:**
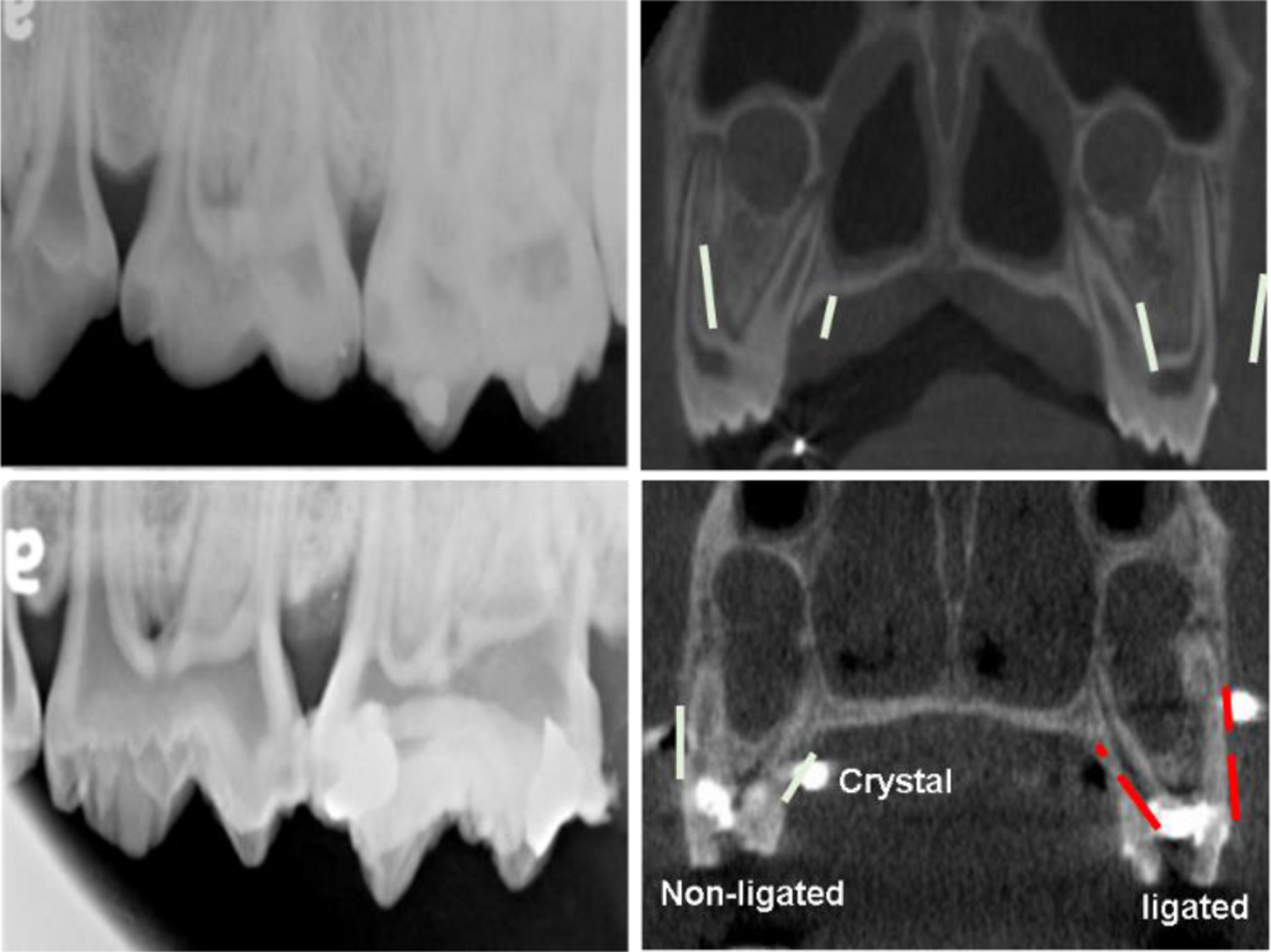
Comparisons of alveolar ridge height between baseline (upper row) and 8 weeks after experimental periodontitis induction (lower row) in both PA (left column) and CBCT images (right column).

The successful induction of experimental periodontitis was verified in both bilateral and unilateral inductions over the period of 8 weeks by the following presentations: 1) formation of significantly deeper (>4 mm) periodontal pockets as compared with controls and adjacent second molar (Figure 2); 2) significant plaque accumulations around the ligated molar with severe bleeding by probing with or without pus discharge (Figure 1B); 3) the alveolar ridge heights around the ligated molar were decreased significantly with the range of 2.7–5.9 mm in both PA dental films and CBCT images, but those around the adjacent molar were not affected and showed only 0–1.5 mm height changes (Figure 3).

Although experimental periodontal disease was successfully induced over time, this disease seemed to have no effect on pigs appetite and general health, because weekly body weight tracking for the PG indicated that the body weight increased over time, leading to a 29–59% gain at the end of the 8 weeks as reported elsewhere [9].

### Harvested tissue processing and image capturing

Upon euthanasia, cardiac perfusion was performed with 0.9% saline solution followed by diluted Prefer, a formalin-free fixative (Anatech Ltd, Battle Creek, Michigan), and the bilateral upper alveolar/molar blocks were harvested. After 1.5–2 weeks of fixation in Prefer solution, the blocks of one side for the bilateral induction or distal half of the target molars for the unilateral deduction were embedded in plastic and sectioned for the examination of alveolar bone loss and mineralization as reported elsewhere [9]. The blocks of the other side or mesial half of the molars were decalcified using the solution of Immunocal (Decal Chemical Corp. Taliman, NY) and embedded in paraffin marked with the mesial-distal orientation. These blocks were sectioned coronally at a thickness of 10μm and stained with either hematoxylin & eosin (H&E) or Sirius Red (SR) in a consecutive order.

**Figure 4:**
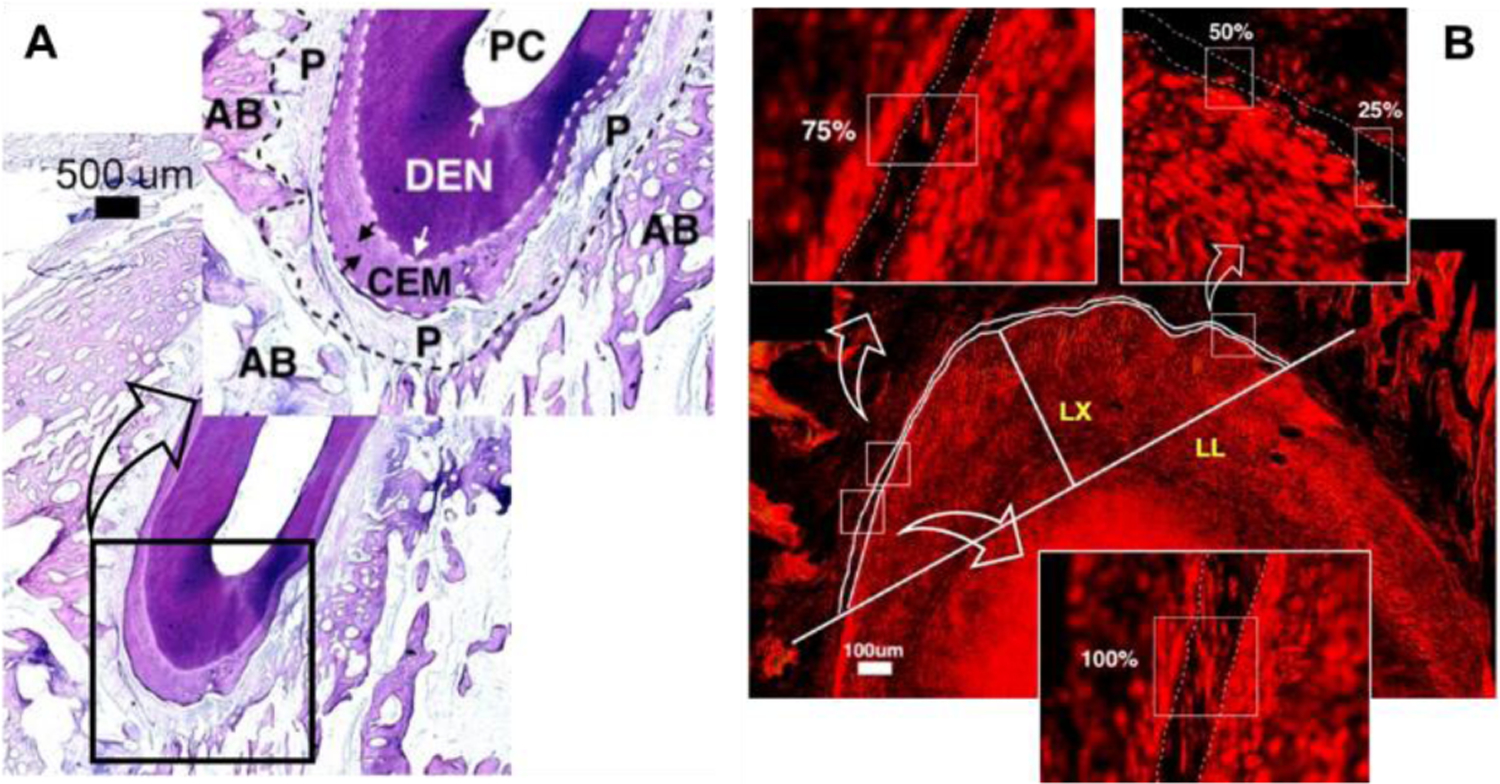
A: Microscopic image of PG #14, H&E stained, 2X; **B:** Polarized image of PG #14. Sirius-Red stained. 10X. **CEM:** Cementum; **DEN:** Dentin; **P**: Periodontal ligament; **PC**: Pulp chamber; **AB:** Alveolar bone. **Line LX**: Long axis of the root; **Line LL**: 500 μm from the apex and perpendicular to the line LX. Three inserts (indicated by the curved arrows) present the enlarged examples of the connection types of the Sharpey’s fiber bundles (100%, 75%, 50%, and 25%).

By use of a Nikon Eclipse E400 microscope equipped with a Spot RT digital camera (Nikon Corporation, Tokyo, Japan), consecutive 4–5 H&E-stained sections across the apex of the mesial palatal root (MPT) were previewed and captured at 2X magnification with the use of Metavue software (Universal Imaging Corporation, Downingtown, PA, USA). After reviewing the structures and alignments of the periodontal ligaments (PDL) to the tooth roots and alveolar bones on these H&E-stained images (Figure 4A), each adjacent SR-stained section was chosen, and the image was captured at the region close to the apex of the mesial root with 10X magnification by using the polarizing light. Due to the birefringent nature of Sirius Red molecule, the collagen fibers stained by this material are brighter and show more contrast with the rest of the tissue under polarized light [7]. Thus, the polarized light was used to image Sirius Red Stained tissue for better presentations of optically anisotropic character of the PDL (Figure 4B).

### Image analyses and statistics

**Figure 5:**
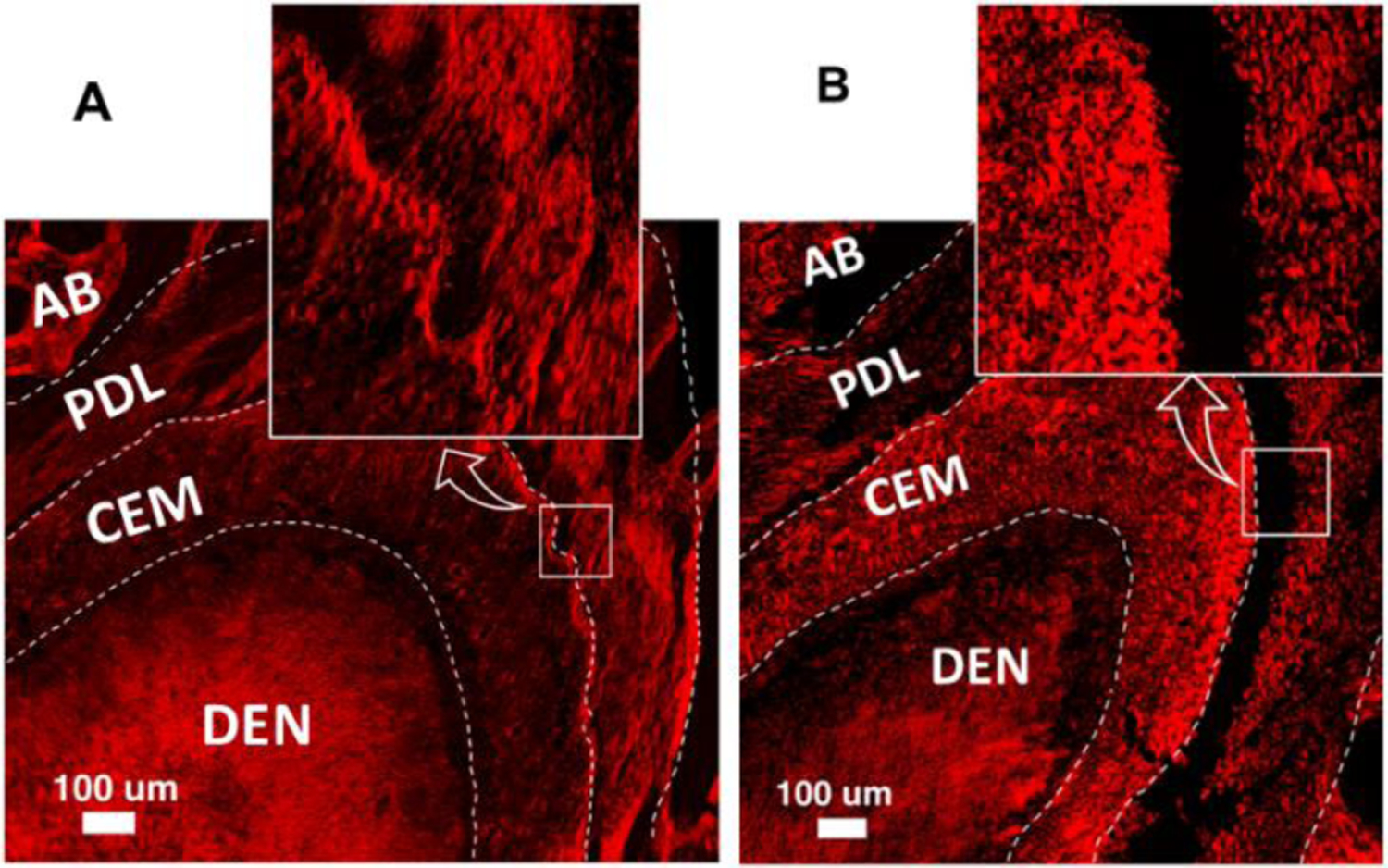
Comparison of the polarized images between the CG (A, #7) and PG (B, #17), 10X. **CEM:** Cementum; **DEN:** Dentin; **PDL**: Periodontal ligament; **AB:** Alveolar bone.

The image analyses were focused on the area 500μm apical to the apex (Figure 5). This region was targeted because the PDL space of the root apex increased more significantly than those of buccal-palatal and mesial-distal sides with the experimental periodontitis [13]. The investigators were blinded to the image assignments. Since certain sections were not well stained with either H&E or SR, a total of 3 bilaterally induced, 3 unilaterally induced and 8 control animals were included in the following image analyses. Before doing image analysis, a space was defined between the cementum and the alveolar bone by manually drawing two lines, one at the cementum side and the other at the bone side. All following analyses were done based on this defined space for each image: 1) the total Sharpey’s bundle numbers; 2) fibers in each Sharpey’s bundle (bundle size); 3) Sharpey’s fiber bundle attachments: defined as the continuation of the bundle in the defined space between the root cementum and alveolar bone. A complete bundle attachment without interruption was categorized as 100% connection, and interrupted connections with different degrees were categorized as 75%, 50%, and 25% connections. These percentages were calculated based upon the measured length of the bundles along the defined space between the cementum and alveolar bone. The 75%, 50% and 25% bundle connections means that the bundle fills > 68.5%, between 37.5% to 68.5%, and less than 37.5% of the defined space, respectively. The sides of the interruptions within the bundles were further recognized as toward the cementum or alveolar sides; 4) angulation of Sharpey’s fiber bundles: taking the long axis of the target molar root, the apical-oriented angle of 5 to 7 randomly selected bundles were measured and averaged. The periodontitis and control sides of 3 unilateral induction animals were analyzed separately, because the so-called control sides could have been infected by the induction as well.

The measurement reliability was tested as follows: 1) inter-investigator reliability: the primary (ME) and secondary (LL) examiners measured the same 10 images, and the two sets of their results were assessed by the correlation coefficient; 2) intra-investigator reliability: the same 10 images were re-measured by these two investigators at the interval of 10 days as well, the results were assessed by Dahlberg equation [14].

Descriptive statistics were used to analyze the data from each group to calculate means, standard deviation, and ranges. Since the skewness test indicated the data is not skewed, independent t-tests were applied for each variable between the two groups. The significance level was set at p ≤ 0.05.

## Results

The reliability tests across all variables show that the t and Dahlberg values were 0.24–0.99 and 0.27 – 7.00 for inter- and intra-investigator tests, respectively, indicating the reliable repeatability of approaches for data analyses in the present study.

**Figure 6:**
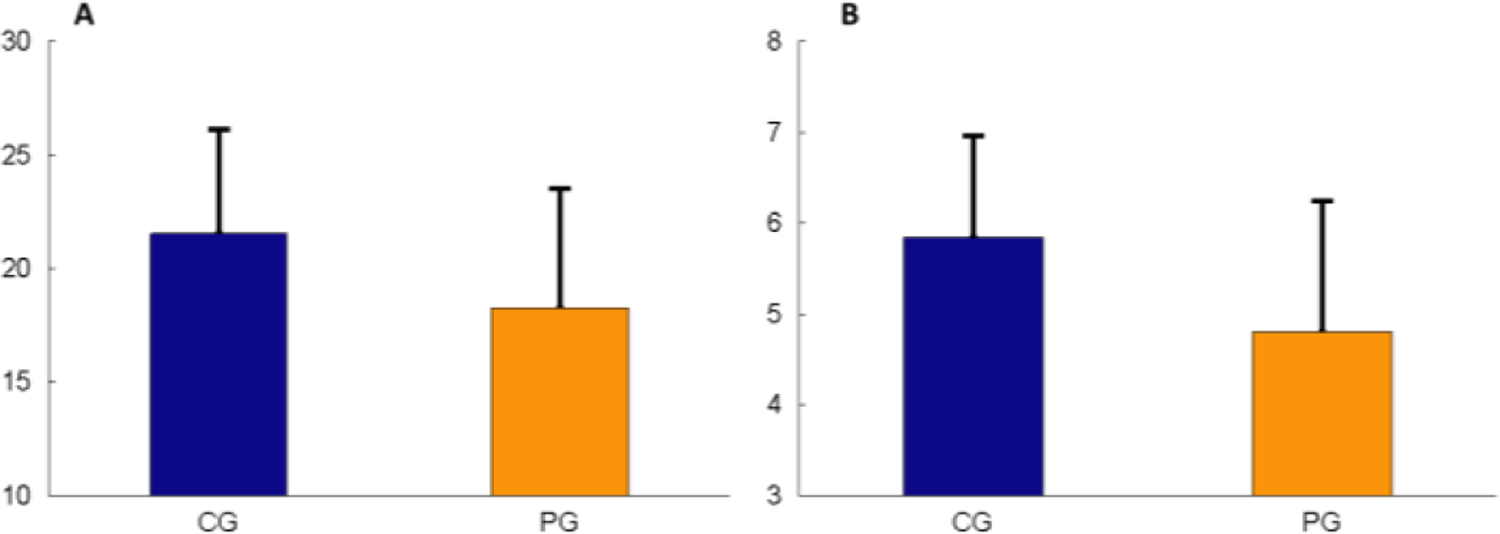
Comparisons of the total bundle count (A) and bundle size (B) between the PG and CG.

Compared the PG to the CG, the total bundle count showed slightly less (Figure 6A) with significantly lower number of 100% connected bundles (p=0.05), and the bundle size was slightly smaller in the PG as well (Figure 6B).

**Figure 7:**
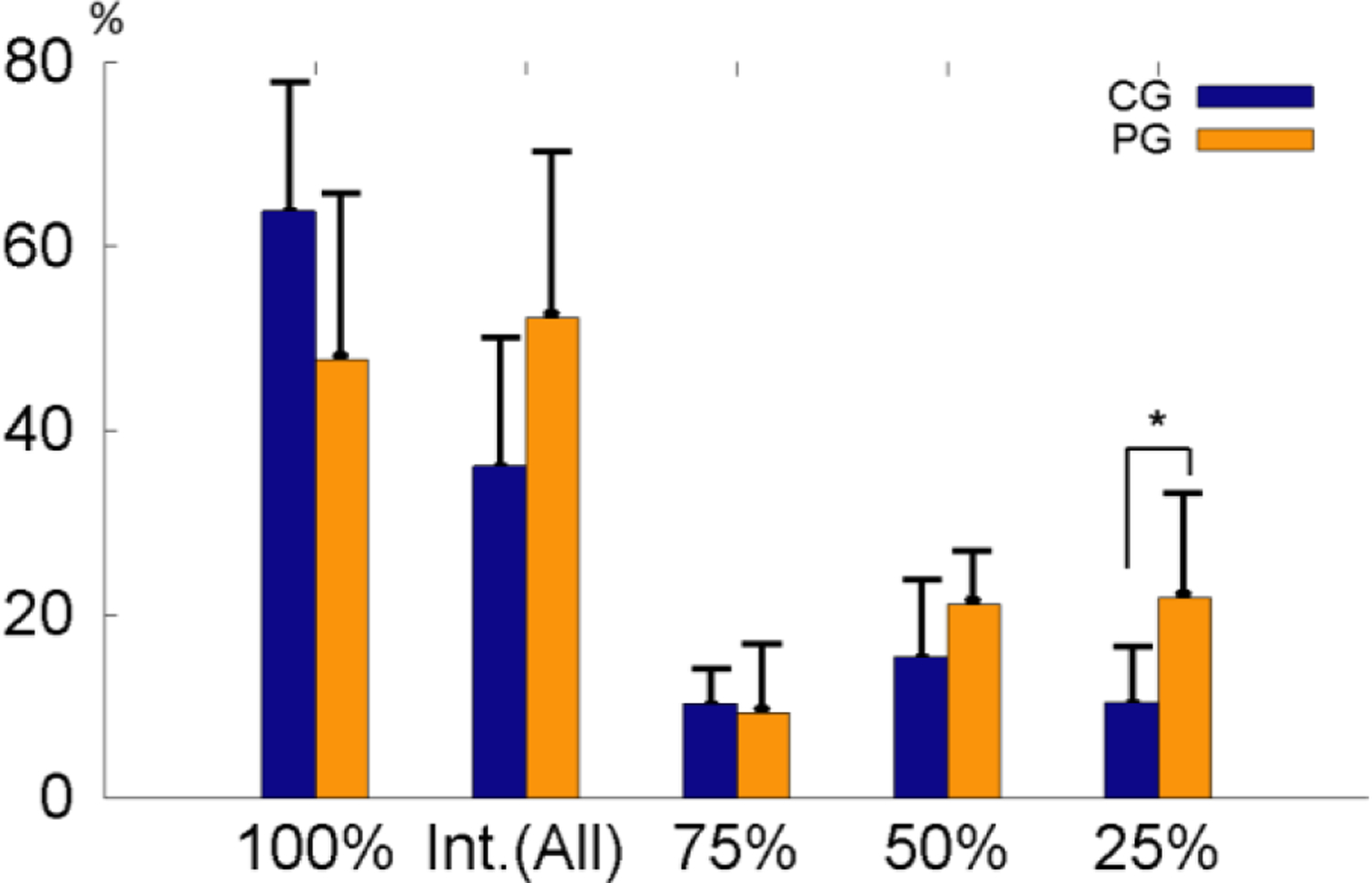
Summaries of the connection types (100%, 75%, 50%, and 25%) between the two group. Int. (All): combination of all interrupted Sharpey’s fiber bundles

**Figure 8:**
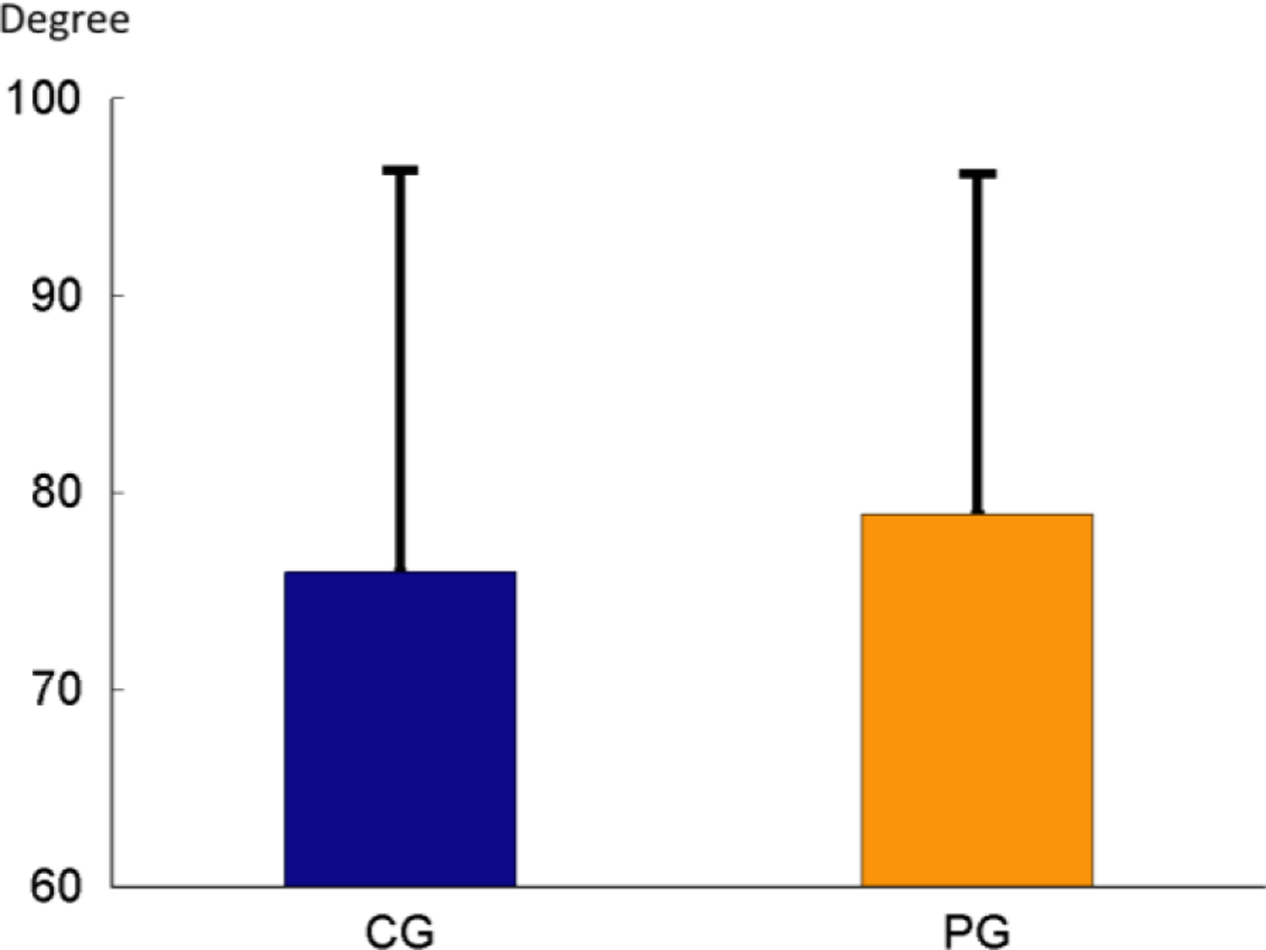
Comparison of Sharpey’s fiber bundle angulation between the two groups.

As shown in (Figure 7), the average % of the 100% bundles was lower but the % of the total interrupted bundles were higher in the PG. When average % of each interrupted bundle type was compared, the 50% and 75% interrupted bundles were slightly higher (p=0.18) and lower (p=0.74) in the PG respectively, but the 25% interrupted bundles was significantly higher in the PG (p=0.03). The bundle angulation oriented more cervically in the PG as compared to the CG (Figure 8).

**Figure 9:**
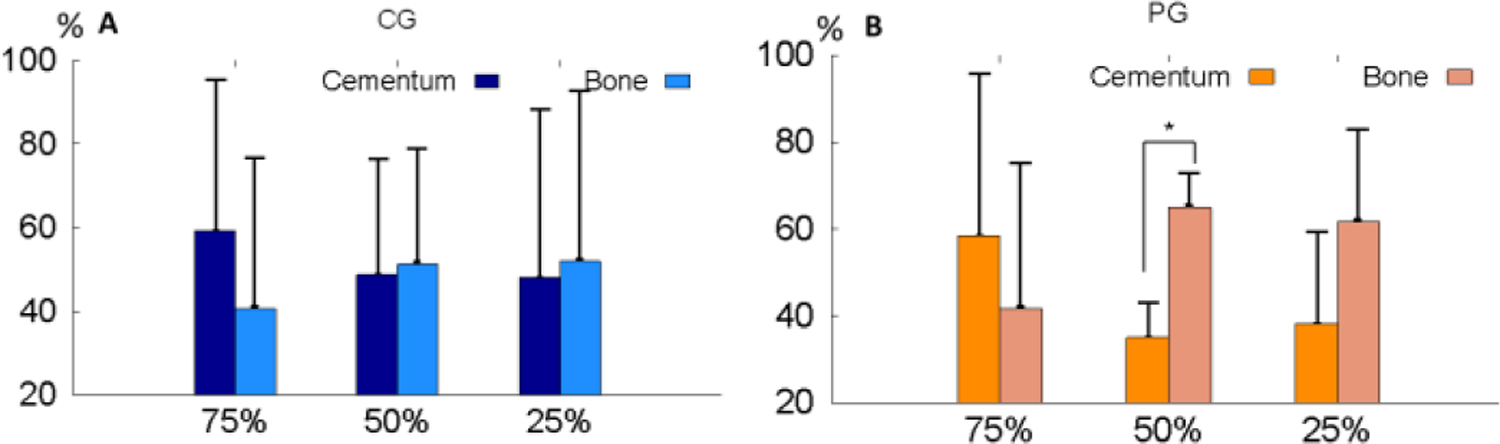
**A:** Average % of bundle interruptions side in CG. **B:** Average % of bundle interruptions side in PG.

When looking into the interrupted sides of the interrupted bundles, root cementum side were seen more in the 75% interrupted bundles, whereas alveolar bone side were more often seen in the 50% and 25% interrupted bundles. These trends looked similar in both PG and CG, but the PG showed larger difference than the CG did (Figures 9A and 9B). The significant difference was found for the 50% bundles (p<0.01).

The results from the 3 animals with unilateral induction were not consistent with the above comparisons. The most significant feature is that the control side showed similar, or even worse Sharpey’s fiber bundles’ presentations as seen in the side with the induced periodontitis. More specifically, the total bundle count in control side of the pig #16 was the lowest among all the animals in this study, and these for control sides of pigs #18 and #19 were closer to the PG. In addition, all the 3 control sides showed lower intact bundle % compared to their periodontitis-inducted sides. In addition, the interrupted bundle % from the control sides were higher compared to their periodontitis-inducted sides. The bundle angulation and interruption side location showed the similar pattern to the main analysis (Table 1).

## Discussion

### Bundle types and Characteristics

The present study confirmed that the number of fully connected Sharpey’s fiber bundles were significantly less in the PG. This strongly suggests that experimental periodontitis might have damaged Sharpey’s fiber bundles. Greater number of connected bundles shows more attachments of PDL to cementum in healthy periodontium [15].

The characteristics of the bundle types are better reflected when each bundle type was summarized (Figure 7). While the percentages of the intact connection bundles (100%) and the total of interrupted bundles were less and more in the PG than CG, respectively, the 75% interruption type looks very similar in the two groups. However, the increase of 50% and 25% interruption types in the PG were more obvious with significantly more for the 25% types. The study on the PDL width on the same animals indicated that the apical PDL width was significantly higher in PG comparing to the CG, and these increased PDL width looks similar to the periodontal abscess [13]. Therefore, these increased PDL width might be resulted from the damages of the Sharpey’s fiber bundles in the apical region.

The opposite results of bundle types from the 3 unilaterally induced pigs could be due to the periodontal bacterial transfered from periodontitis side to the control side. As a result, the bacterial presence could have some effect on the control side molars without showing clinical inflammation. The measurements of the apical PDL widths on these unilateral induced animals further support this possibility because the width was significantly higher in both control sides and periodontitis sides as compared with the CG [13].

### Bundle size and angulation

The studies have indicated that distribution of Sharpey’s bundles varies with the regions [7], and more functional loads lead to more bundle density [16]. However, the present study only examined the apical region. Thus, no region difference could be addressed. To the best of our knowledge, Sharpey’s bundle size has not been documented in the literature. The present study found that each normal Sharpey’s bundle in young pigs contains 4–7 principal fibers, and this number was slightly lower in the PG (Figure 6B). This finding should be considered as preliminary because not all observed bundles were counted due to the technical difficulty as described in the Materials and Methods.

There is an approximate 5–15° difference between the embedding angle (acute angle between the Sharpey’s fibers and the bone/PDL or cementum/PDL interfaces inside bone or cementum) and the insertion angle (acute angle between the PDL fibers and the bone/PDL or cementum/PDL interfaces in PDL) of the Sharpey’s fibers in rats [7]. According to some recent investigations, the nature of the PDL is more found to be anisotropic, mostly due to the collagen fibers [17]. This non uniformity is for better tissue function in the presence of loads and is not a response to pathological factors [18]. In the present study, we found the Sharpey’s fibers around the apex oriented anisotropically as well. The average apical angulation of the Sharpey’s fiber bundles was between 60 to 80 degrees. This range of the angulation is consistent with some report in rats and genuine pigs [19], and the angulation was more toward cervical in the PG animals. This result may indicate the experimental periodontitis not only lead to the damages of structures of PDL fibers, but also alter the orientation of Sharpey’s fiber bundles. Both structural and angular changes in the PG animals might be resulted from the periodontitis itself, or from the changes of functional loading on the teeth due to more mobility in the socket [20].

### Sides of bundle interruption

A rat study indicated that the Sharpey’s fibers are more dense in the cemental than alveolar bone sides, but the diameter of the Sharpey’s fibers are two times greater on the bone than the cemental sides [7]. In the present study, the overall differences between the locations of the bundle interruptions were not significant between the two groups except for 50% bundles in the PG where the interruptions occurred significantly more in the bone than the cemental sides (Figure 9). Both CG and PG presented the similar pattern of the interruption: more on the cemental side for 75% bundles and more on the bone sides for 50% and 25% bundles. Such side preference of the Sharpey’s fiber interruption were seen in both sides of the unilaterally induced animals. Overall, the interruption of Sharpey’s fibers in the apical region occurs more in cemental than bone sides, regardless of healthy or diseased condition.

## Limitation of the study

A pig model was chosen due to its relative similarity to humans, including both the comparable size, morphology, and function of the jaw and teeth in contrast to other middle- or large- sized animal models such as rabbit or sheep [21]. However, relatively small sample sizes are the major limitation of this study. This was due to distortions that occurred during the process of histological slide preparation and staining. As a result, it was difficult to capture good quality images on some of these slides, which eventually led to their removal from data analysis.

The inclusion of the unilaterally induced animals might add the limitation of the present study, and the investigation restricted in the apical region could further limit the scope of this study. The other limitation is that it is uncertain whether every individual bundle in the defined areas of polarized images was analyzed for the bundle size and orientation due to some blur presentations in the polarized images.

The last limitation of this study is that the deciduous rather than permanent molars were used for the study. There are the two major reasons behind this approach: 1) the permanent molar erupts distal to the last deciduous molar in pigs thus the deciduous molar PDL is not affected by permanent molar eruption; and 2) the pig is very difficult to handle due to the size and weight when reaching the age with the full eruption of the permanent molar (about the age of 10–12 months). Nevertheless, to the best of our knowledge, this is the first study to quantify and characterize Sharpey’s fibers in the PDL with normal and experimental periodontitis in a large animal model.

## Conclusions

The present study confirmed that experimental periodontitis damages PDL structures in the apical area in young pigs’ molar compared those in healthy controls. These damages include: 1) increased numbers of interrupted Sharpey’s fibers, particularly in 25% interruption type; 2) reduced size/density of the Sharpey’s bundles; and 3) altered orientation of the Sharpey’s bundles. Given the limited examinations in the apical region of the target molar only, the future study may need to extend to the entire PDL spaces in different teeth.

## Figures and Tables

**Table 1:** Comparison of all variables between inducted and non-inducted teeth in three unilaterally induced (split mouth) pigs

Pig#	Total bundle		Intact (100%) bundles		Interrupted bundles		Bundle size		Bundle angulation	
	Non-inducted	Inducted	Non-inducted	Inducted	Non-inducted	Inducted	Non-inducted	Inducted	Non-inducted	Inducted
**16**	6	21.67	16.67	62.18	83.33	37.82	3.25	3.68	41.33	70.9
**18**	12	16.33	14.14	34.72	85.86	65.28	4.4	5.5	99.38	47.83
**19**	19	15	37.88	77.25	62.12	22.75	4.46	5.99	55.5	82.16
SD	6.51	3.53	13.04	21.56	13.04	21.56	0.68	1.22	30.27	17.5

SD: Standard Deviation
